# The revolving door of HIV care: Revising the service delivery cascade to achieve the UNAIDS 95-95-95 goals

**DOI:** 10.1371/journal.pmed.1003651

**Published:** 2021-05-24

**Authors:** Peter Ehrenkranz, Sydney Rosen, Andrew Boulle, Jeffrey W. Eaton, Nathan Ford, Matthew P. Fox, Anna Grimsrud, Brian D. Rice, Izukanji Sikazwe, Charles B. Holmes

**Affiliations:** 1 Global Health, Bill & Melinda Gates Foundation, Seattle, WA, United States of America; 2 Department of Global Health, Boston University School of Public Health, Boston, MA, United States of America; 3 Health Economics and Epidemiology Research Office, Department of Internal Medicine, School of Clinical Medicine, Faculty of Health Sciences, University of the Witwatersrand, Johannesburg, South Africa; 4 School of Public Health and Family Medicine, University of Cape Town, Cape Town, South Africa; 5 MRC Centre for Global Infectious Disease Analysis, School of Public Health, Imperial College London, London, United Kingdom; 6 HIV & Global Hepatitis Programme, World Health Organization, Geneva, Switzerland; 7 Centre for Infectious Disease Epidemiology and Research, School of Public Health and Family Medicine, Faculty of Health Sciences, University of Cape Town, Cape Town, South Africa; 8 Department of Epidemiology, Boston University School of Public Health, Boston, MA, United States of America; 9 HIV Programmes & Advocacy Department, International AIDS Society, Cape Town, South Africa; 10 Department of Public Health, Environments and Society, Faculty of Public Health and Policy, London School of Hygiene & Tropical Medicine, London, United Kingdom; 11 Centre for Infectious Disease Research in Zambia, Lusaka, Zambia; 12 Center for Innovation in Global Health, Georgetown University, Washington, DC, United States of America

## Abstract

Peter Ehrenkranz and co-authors present a cyclical cascade of care for people with HIV infection, aiming to facilitate assessment of outcomes.

**Summary points**

- Antiretroviral therapy (ART) for human immunodeficiency virus (HIV) prevents illness and death from HIV disease and transmission of HIV infection. To encourage global scale-up of ART, the Joint UN Program on HIV/AIDS (UNAIDS) issued the “95-95-95” targets for the HIV “cascade of care.” These targets state that by 2030, 95% of individuals living with HIV will know their HIV status, 95% of people with diagnosed HIV infection will receive ART, and 95% of those taking ART will have achieved suppression of the virus.
- While tremendous progress has been made toward achieving these targets, substantial gaps remain. The challenge of closing the final gaps requires reconsideration of the cascade itself.
- The 95-95-95 HIV care cascade depicts a linear and unidirectional continuum of care with one starting point (HIV diagnosis) and one ending point (treatment discontination or death). This simplification of the cascade oversimplifies the complex cycle of engagement, disengagement, temporary disuptions, reengagement, and transitions in care experienced by many people living with HIV (PLHIV).
- As the proportion of PLHIV who reinitiate ART after previously starting and stopping increases, we propose to update the HIV cascade of care to better reflect actual experiences of PLHIV. The new cascade makes the cycle of engaging and reengaging in HIV care both explicit and expected.
- The revised cascade will inform and prioritize efforts by communities, healthcare workers, implementers, program managers, policymakers, and donors to prevent missed clinic visits, overcome barriers to care reentry, and minimize onset of advanced HIV disease. It will also emphasize that morbidity, mortality, and onward transmission can be minimized by focusing interventions on anticipating, and then reducing, the duration of gaps in care.

## Introduction

Since 2014, the global public health community has recognized a set of targets for human immunodeficiency virus (HIV) known as “90-90-90,” an ambitious plan that called for the diagnosis of 90% of people living with HIV (PLHIV), antiretroviral therapy(ART) for 90% of those diagnosed HIV–positive, and viral suppression in 90% of those receiving ART by 2020 [1]. In an effort to end HIV as a global health threat, these initial goals were extended to achieve “95-95-95” by 2030 [2]. Despite tremendous progress toward achieving these objectives, challenges remain. At the end of 2019, an estimated 81% of PLHIV globally knew their HIV status, 82% of these were on ART, and 88% of people on ART were virally suppressed, suggesting an overall viral suppression proportion of just 59% [3].

To understand why countries continue to fall short of achieving the 95-95-95 targets, one must understand the frequency and circumstances within which people enter and leave HIV care. While PLHIV continue to disengage from care between HIV diagnosis and ART initiation [4], the recent push for rapid, including same-day, ART initiation, which essentially eliminates losses from care before ART initiation, is leading to a shift of interruptions in care to a point further down the cascade [5]. This effect becomes even more pronounced during periods of major service disruption through conflict, natural disaster, or epidemics [6]. Population-level control of HIV will remain out of reach if many people initiating treatment disengage from ART for long periods of time, as they will have increased opportunity for viral load failure, morbidity, mortality [7], development of drug resistance, and viral transmission [8–11].

Cascades have become a common approach to measuring engagement and outcomes of public health programs and assisting in prioritizing interventions. Most HIV care cascades depict a linear, unidirectional continuum in which a person enters at the beginning and only exits upon death or loss to follow-up. This representation has been helpful to compare progress between geographies and populations and to identify challenges to continuity of care [12]. Multiple studies in sub-Saharan Africa, however, have documented misclassifications of both the numerators and denominators within these cascades. Some people who appear to have disengaged actually remain in care at the same facility or have “silently” transferred to another (meaning they do not inform the initial clinic that they are leaving or the receiving clinic that they were a recipient of care elsewhere). Others have died [7,13–16]. In addition to misclassification challenges, linear cascades risk oversimplifying the complex cyclical cycle of entry and reentry into care experienced by many PLHIV. These simplifications may result in failure to detect immunosuppression or emergent drug resistance resulting from transient treatment interruptions and inaccurate prioritization of interventions aimed at improving long-term retention and viral suppression [17].

Many PLHIV start and stop ART multiple times over the course of their lives, creating what has been termed a “side door” into the cascade through which individuals who have left the system reenter it [18]. Some people report fear of being treated poorly if they return to care after an absence [19] and may perceive it as more acceptable to retest and restart ART at a new facility as a “new” patient rather than facing censure from healthcare staff [20,21]. Routinely collected medical records in most settings do not adequately document this phenomenon. In one notable exception, South Africa’s Western Cape Province undertook a pilot that used unique patient identifiers and digitized routinely collected point-of-care HIV test results to assess testing and restarts. Within their intervention site, 51% of people who tested HIV–positive had previously been diagnosed, and 71% of these had previously started ART. In other words, more than one-third of HIV testers had previously been on ART [22]. This information was used to highlight the need to redirect resources from expansion of HIV testing to improved focus on continuity of care.

With an increasing proportion of initiators being non-naive to ART and the growing implementation of same day ART initiation, we propose the introduction of an HIV cascade of care that better captures the nonlinear HIV journey and defines the numerator and denominator at each step. This revision can support stakeholders, including Ministries of Health, providers, and donors, to recognize that while treatment interruptions may be inevitable for some people over a lifetime of HIV treatment, negative public health and clinical consequences could be minimized by focusing on decreasing gaps in care. Being “disengaged” or “engaged” is not a final state: It is an interval within the lifetime of a recipient of care. A cascade that documents actual PLHIV behavior—the cycle of engaging and reengaging in care—would inform and prioritize efforts intended to prevent missed clinic visits, overcome barriers to reentry, and minimize onset of advanced HIV disease [5,23–25].

## Proposal for a cyclical cascade

**Fig 1:**
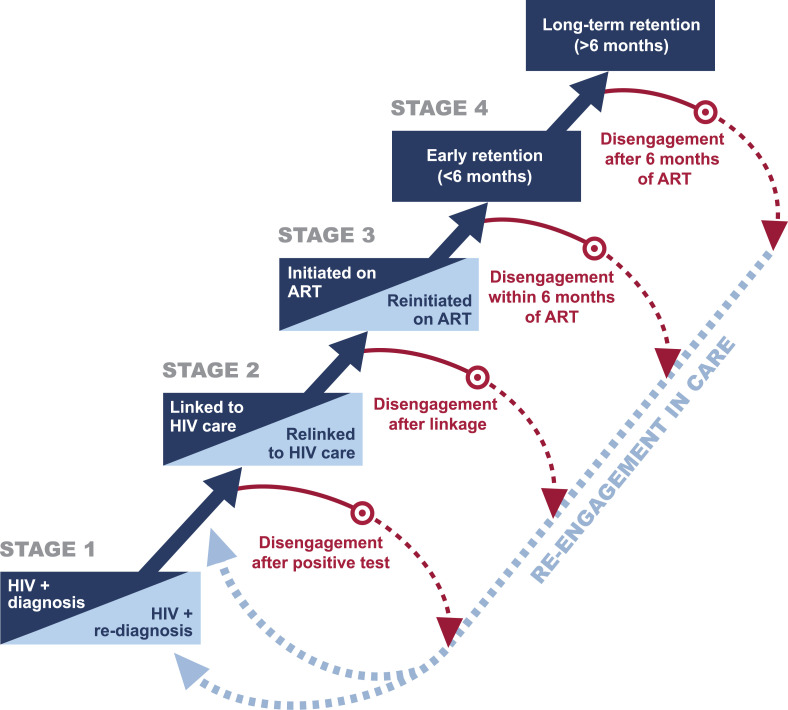
Cyclical cascade of HIV care. ART, antiretroviral therapy; HIV, human immunodeficiency virus.

To capture the care pathways of PLHIV, we propose a cyclical cascade as illustrated in **Fig 1** and defined in **Table 1**. Following prior cascades [17], the proposed cascade defines four linear stages plus a stage of disengagement that is an alternative path after each stage. Reengagement is represented as a dotted line as it is a transition state between disengagement and resumption of service delivery.

**Table 1:** Definitions of the stages of the cyclical cascade of HIV care.

**Stages of the cyclical HIV cascade**	**Definition**
Stage 1: HIV+ diagnosis/HIV+ re-diagnosis^a^ ➔ Linked/relinked	The interval from receiving an HIV–positive diagnosis to enrollment in an HIV treatment program as a new or returning client
Stage 2: Linked/relinked ➔ Initiated/reinitiated^b^	The interval from enrollment in an HIV treatment program as a new or returning client to receiving ART
Stage 3: Initiated/reinitiated ART➔ Early retention (until first viral load test result received or maximum of 6 months after ART start)^c^	The interval from first dose of ART to initial viral load test result, which the 2021 WHO guidelines strongly recommend be reviewed by 6 months after initiating ART [26]
Stage 4: Early retention➔ Long-term retention (beyond first viral load test, often after 6 months)	The interval from initial viral load test (currently, most national guidelines recommend after 6 months on ART) to final disengagement from care and/or death
Disengagement^d^	A gap of >30 days without taking ART

^a^“HIV+ re-diagnosis” denotes the situation in which a person who is aware of their HIV diagnosis and who has interrupted care at any point in their treatment journey uses testing as an opportunity to reengage with care. This usage is distinct from recommendations for people who are HIV–negative to repeat testing at regular intervals determined by their risk status.

^b^Many PLHIV may go directly from testing to same day ART initiation (complete Stages 1 and 2 in 1 day). However, there remains an opportunity for disengaging between Stages 1 and 2 that warrants continuing to define 2 stages (with self-testing as well as with more traditional testing modalities), at least until there are sufficient data to demonstrate that no gap exists between these 2 stages.

^c^In the future, Stage 3 may decrease in duration if, for example, time to first viral load test is reduced to 4 months after initiation with new first-line regimens.

^d^While a gap in ART adherence of more than a few days may have clinical consequences and a gap of 7 days in appointment keeping should prompt tracing efforts, we propose 30 days as an indication of a change in care behavior significant enough to be considered “disengagement.” This time period may need to be adjusted with implementation of long-acting injectable ART or local preference.

Updated from [17].

The primary innovation of the proposed cascade is the inclusion of potential disengagement at each of the four stages and opportunities for reengagement at the first two: (re)diagnosis and (re)link. By explicitly capturing PLHIV revolving into and out of each of the stages of care, it becomes possible to both pose and answer novel questions. While a linear cascade can describe the stage at which PLHIV are most likely to exit treatment, it cannot answer the following: (1) What are the stages in the revised cascade with the most reentries back into the health system? (2) What is the frequency of repeat exit and reentry? (3) Which stages most correlate with return without intervention versus as a result of an intervention? (4) What are the implications of loss/reentry at a given stage on future losses? Failure to answer these questions can impede our ability to develop effective interventions to support continuity of care and effectively utilize available resources.

While scale-up of same-day ART initiation in many facility or community settings may lead to the merging of Stages 1 and 2, the proposed cascade maintains linkage/re-linkage as a separate intermediate step. We define linkage or “enrollment” as assignment of a unique person number and/or establishment of a medical record with the intention of prescribing ART. If a person is identified as having reengaged with care or has an existing health system number, they may be reassigned their original number and/or record, but, practically speaking, they will often be assigned a new one. As programs may have different definitions for “enrollment,” it may be challenging to compare this indicator across countries. However, a localized version could be used to measure the extent of disengagement that occurs between diagnosis and initiation over time. We are also proposing retention be split into “early” (first six months or time to first viral load test result) and “long-term” (after first viral load test result or first six months on ART) as there are important differences in service delivery between these time periods. The proposed definition for where Stage 3 ends and Stage 4 begins is based on the updated 2021 WHO guidance, which “encourages that the first viral load result be … reviewed by 6 months after initiating ART” [26]. This interval should be adjusted as evidence accumulates or as guidance on timing of first viral load test changes. Clinical outcomes, such as viral load suppression, are not explicitly included in the cascade as long-term retention in care, the fourth stage in our cascade, is highly correlated with suppressed viral load [7,27], and our intention is to better understand PLHIV behavior as it relates to patterns of engagement, not the biological results of treatment. Finally, the proposed cascade does not identify the facility at which people return to care or indicate how long they were disengaged. These data points are critical, however, and will require additional investigations.

## The cyclical cascade can help target interventions

By embracing the cyclical nature of engagement with HIV care, programs can use resources more effectively. Research can uncover the demographic and clinical characteristics (including comorbidities), needs, behaviors, perceptions, and preferences of PLHIV most at risk of disengagement and their communities’ influence on their continuum of care. With this information, targeted (and, optimally, generalizable and scalable) interventions can be introduced that will (1) identify people at greatest risk of disengagement and help support their retention in care (i.e., minimize disengagement); (2) rapidly detect people who appear to have disengaged, confirm their status, and facilitate their return to care as needed (i.e., reverse disengagement that has already occurred or identify silent transfers and remove duplicate charts within the record system); and (3) target different interventions to individuals depending on whether they have been in a stage of the cascade before. Such work has already been conducted in Zambia [28,29] and South Africa [30] and could provide a foundation for efforts in other settings.

Identifying which PLHIV are at greatest risk of disengagement from within subpopulations is a high priority. Population HIV impact assessments (PHIAs) in five southern African countries have reported that among people with nonsuppressed viral load, marriage, female sex, shorter ART duration, higher CD4 count and alcohol use were associated with higher odds for interrupted ART [31]. Many may have been women who were offered rapid ART initiation during an antenatal program following Option B+ guidelines. However, most individuals, even within these high-risk groups, are not likely to interrupt treatment. Targeting the entire population group, without identifying the minority who are truly at risk, is an inefficient use of resources. For example, “welcome back” programs have been introduced in some settings [33], but there has been little effort to direct such interventions more precisely toward individuals most likely to disengage, including those with a history of disengagement.

## Populating and using the cyclical cascade

Utilizing the proposed cyclical cascade as a framework for supporting program management will require data that are challenging to collect at a routine, programmatic level. People who discontinue care are often difficult to trace, but there are a few accurate reports of the proportions of ART initiators who were previously on treatment that include their characteristics or reasons for previous default and what it would take for them to return to care [28,33]. Individual-level data like these that cover the entire cyclical cascade are required to develop and target interventions that will minimize exit and facilitate reentry along the continuum of care. Populating the cascade will require multiple data sources, including routine clinical data, surveillance data, and specialized surveys. Of critical importance will be unique national identifiers that enable programs using electronic medical records (EMRs) to track individuals over time along their journey and across all the facilities or community-based programs they may attend, as recommended by WHO [34]. The lack of such identifiers has limited many previous attempts to understand PLHIV movements within health systems and obstructed attempts to provide high-quality person-centered care. In an attempt to answer one such question, a recent analysis of household survey and HIV testing program data across sub-Saharan Africa estimated that “58% of positive tests will have been done on previously diagnosed PLHIV in sub-Saharan Africa in 2020” [35]. If this trend is further validated, efforts to retain and reengage people who have interrupted treatment will become a higher yield activity than population-wide testing to make new HIV diagnoses.

Once data are in hand, interventions can be designed to context in a way that has not previously been feasible. For example, one HIV program might recognize that retesting for HIV (Stage 1) is the most acceptable way for PLHIV who have disengaged from care to reengage and begin to actively encourage it as a pragmatic approach to reinitiating ART. Another program might find that its major challenge is linkage from diagnosis to treatment initiation (Stage 2) among young men and conduct further studies to identify critical structural challenges such as transportation. This program may decide that community-based ART initiation and dispensing medicines covering longer durations are the most promising way to address this issue [36]. A third program may identify missed clinic visits during the first six months after initiation (Stage 3) as the critical predictor of disengagement; these latter programs could then focus on how to optimize early retention for PLHIV with the characteristics of those most likely to drop out in Stage 3. Further, this new cascade may help motivate the design of interventions beyond those currently included in WHO guidelines (**Table 2**) [37] or described in the published literature [5] as new population needs are identified.

**Table 2:** WHO evidence-based recommendations and good practice statements to strengthen the cascade.

HIV testing [38]	• Demand creation • Multiple testing approaches (facility-based HTS, community-based HTS, self-testing) • Provider-assisted referral • Social network–based approaches
Linkage to care [38]	• Streamlined interventions to reduce time between diagnosis and engagement in care, including (i) enhanced linkage with case management; (ii) support for HIV disclosure; (iii) patient tracing; (iv) training staff to provide multiple services; and (v) streamlined and colocated services (moderate-quality evidence) • Peer support and navigation approaches for linkage • Quality improvement approaches using data to improve linkage
Initiation of ART [26,34,37,39]	• Out-of-facility ART initiation • Rapid ART initiation, including same-day start • Tailored patient education, counseling, and support to improve uptake of same-day start • Task sharing and decentralization
Retention [26,37,39]	• Offer of 3–6 monthly clinic visits and ART refills, preferably every 6 months if feasible • Package of community-based interventions • Adherence clubs • Extra care for high-risk people • People-centered practices and communication to improve the relationships between patients and healthcare providers
Reengagement [37]	• Tracing and support for reengagement

As depictions of the cascade become more realistic, they will also become more geographically and population specific. A cyclical cascade can focus attention on exactly who is lost, when, where, and why—thereby allowing targeted interventions. At the same time, data from one setting may become even less generalizable than they have been in the past. Facilities that serve large numbers of migrant workers, for example, are likely to see very different patterns of disengagement and reengagement than those that serve more settled populations. It will thus be incumbent on researchers and program evaluators to understand and note the local characteristics of PLHIV behavior and tailor recommendations according to best practices, many of which were defined in a recent review [5].

Finally, an additional benefit of the revised cascade may be that efforts to populate it will result in identifying a potentially large group of people who have disengaged from and then reengaged in care but are still counted as “lost” by sites reporting their outcomes. We may find that ART programs have been more effective at preventing HIV mortality and transmission than past estimates that relied on linear cascades have implied [18].

## Conclusions

HIV programs globally, and their stakeholders and funders, have begun to recognize that the natural course of HIV care is that many people will, at some point or points, disengage from treatment as their preferences, needs, and behaviors change. These same PLHIV may then reengage after a brief or lengthy interval, while a small proportion may never reengage. In this respect, HIV care is likely similar to care of other chronic diseases. Health systems must be equipped to recognize and anticipate this revolving door of HIV care and focus on minimizing the frequency and duration of periods of disengagement. Achieving this shift should start with development and use of a revised representation of a cascade that recognizes the actual ways that PLHIV interact with care. Maximizing retention across the cascade will require recognizing the factors leading to disengagement—structural, clinic based, and individual—and ensuring that they are systematically addressed by providers, communities, and the health system. While a conventional linear cascade remains useful in identifying obvious programmatic challenges and tracking progress toward global targets, a cyclical cascade that acknowledges an individual’s true experience is required to sustain progress and improve outcomes.

## Abbreviations

ART: antiretroviral therapy
EMR: electronic medical record
HIV: human immunodeficiency virus
PHIA: population HIV impact assessment
PLHIV: people living with HIV
UNAIDS: Joint United Nations Program on HIV/AIDS
